# 1-[(4-Hy­droxy­anilino)methyl­idene]naphthalen-2(1*H*)-one

**DOI:** 10.1107/S160053681303451X

**Published:** 2014-01-04

**Authors:** Safia Chahmana, Fatiha Benghanem, Saida Keraghel, Ali Ourari

**Affiliations:** a Laboratoire d’Electrochimie, d’Ingenierie Moléculaire et de Catalyse Redox, Departement de Génie des Procédés, Faculté de Technologie, Université Ferhat Abbas Sétif, Algeria

## Abstract

The title Schiff base, C_17_H_13_NO_2_, crystallizes in the zwitterionic form and an N—H⋯O hydrogen bond closes an *S*(6) ring. The dihedral angle between the aromatic ring systems is 15.62 (9)°. In the crystal, O—H⋯O hydrogen bonds link the mol­ecules into *C*(11) chains propagating in [010].

## Related literature   

For the tautomeric and photochromic properties of Schiff bases, see: Ünver *et al.* (2002 ▶); Blagus *et al.* (2010 ▶); Alpaslan *et al.* (2011 ▶). For related structures, see: Özek *et al.* (2004 ▶); Odabaşoğlu *et al.* (2004 ▶); Yüce *et al.* (2004 ▶).
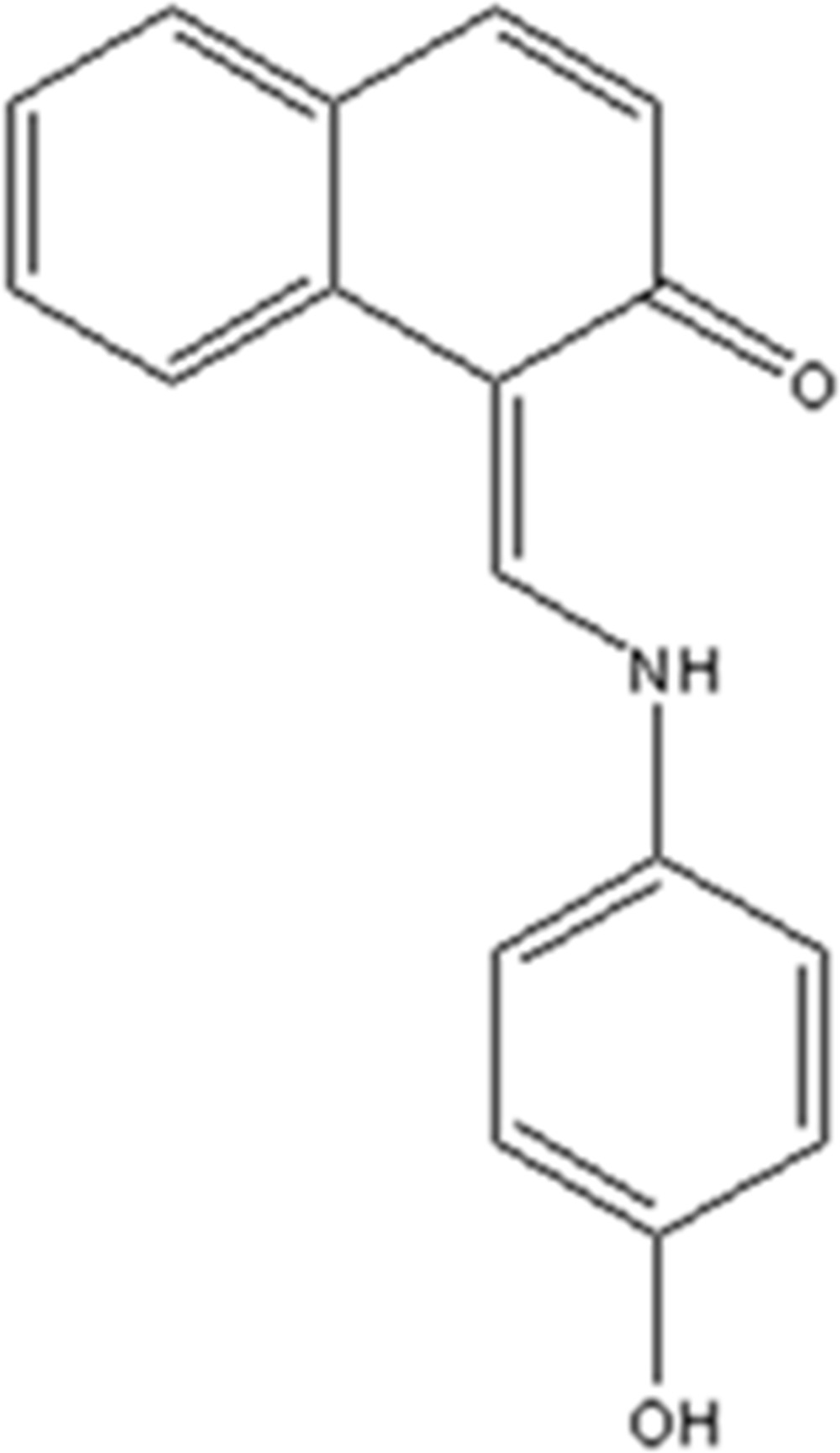


## Experimental   

### 

#### Crystal data  

C_17_H_13_NO_2_
*M* *_r_* = 263.28
Orthorhombic, 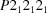

*a* = 6.1997 (7) Å
*b* = 12.9145 (15) Å
*c* = 16.5910 (19) Å
*V* = 1328.4 (3) Å^3^
*Z* = 4
Mo *K*α radiation
μ = 0.09 mm^−1^
*T* = 296 K
0.2 × 0.05 × 0.03 mm

#### Data collection  

Bruker SMART APEXII CCD diffractometer
Absorption correction: multi-scan (*SADABS*; Bruker, 2004 ▶) *T*_min_ = 0.685, *T*_max_ = 0.746
13010 measured reflections
1791 independent reflections
1445 reflections with *I* > 2σ(*I*)
*R*_int_ = 0.038

#### Refinement  

*R*[*F* ^2^ > 2σ(*F* ^2^)] = 0.036
*wR*(*F* ^2^) = 0.099
*S* = 1.11
1791 reflections
189 parameters
H atoms treated by a mixture of independent and constrained refinement
Δρ_max_ = 0.13 e Å^−3^
Δρ_min_ = −0.13 e Å^−3^

### 

Data collection: *APEX2* (Bruker, 2004 ▶); cell refinement: *SAINT* (Bruker, 2004 ▶); data reduction: *SAINT*; program(s) used to solve structure: *SHELXS97* (Sheldrick, 2008 ▶); program(s) used to refine structure: *SHELXL97* (Sheldrick, 2008 ▶); molecular graphics: *ORTEP-3 for Windows* (Farrugia, 2012 ▶); software used to prepare material for publication: *publCIF* (Westrip, 2010 ▶).

## Supplementary Material

Crystal structure: contains datablock(s) global, I. DOI: 10.1107/S160053681303451X/hb7165sup1.cif

Structure factors: contains datablock(s) I. DOI: 10.1107/S160053681303451X/hb7165Isup2.hkl

Supporting information file. DOI: 10.1107/S160053681303451X/hb7165Isup3.cml

CCDC reference:

Additional supporting information:  crystallographic information; 3D view; checkCIF report

## Figures and Tables

**Table 1:** Hydrogen-bond geometry (Å, °)

*D*—H⋯*A*	*D*—H	H⋯*A*	*D*⋯*A*	*D*—H⋯*A*
N1—H2*A*⋯O2	0.98 (3)	1.75 (3)	2.563 (2)	138 (2)
O1—H1*A*⋯O2^i^	0.89 (3)	1.80 (3)	2.680 (2)	171 (3)

Symmetry code: (i) 
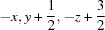
.

**Fig. 1.**
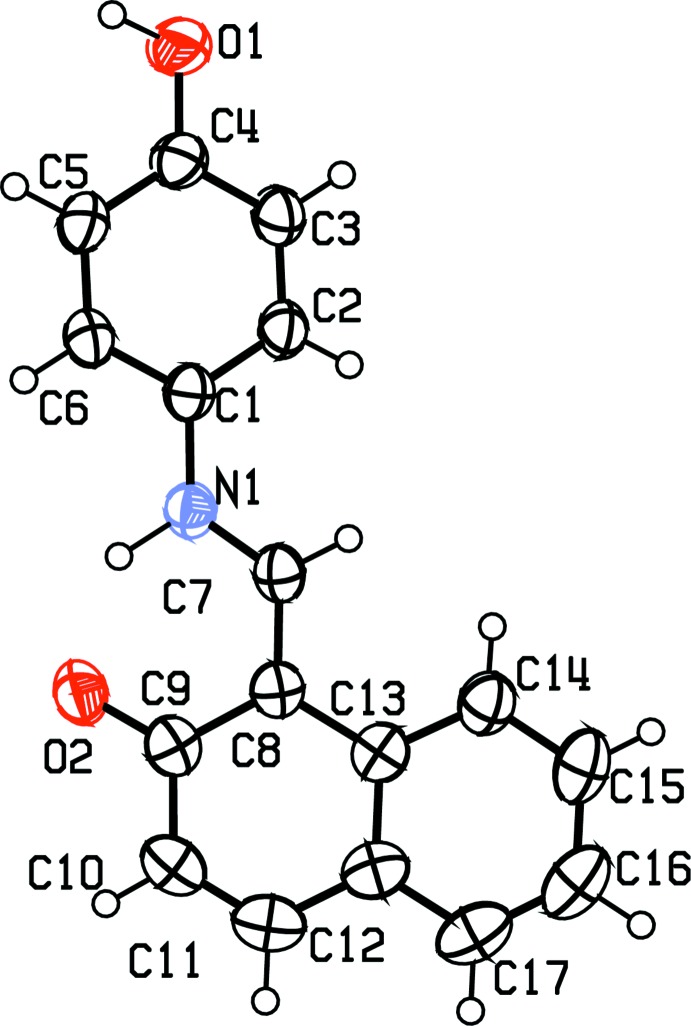
A view of the molecular structure of the title molecule, with displacement ellipsoids drawn at the 50% probability level.

## supplementary crystallographic information

### 1. Comment

The tautomerism of Schiff bases has been studied by (Alpaslan *et al.*, 2011; Blagus *et al.*, 2010; Ünver *et al.*, 2002). It demonstrated that the stabilization of the Keto-amino tautomer in the crystal depend mostly on the parent *o*-hydroxyl aldehyde, the type of the N-substituent, the electron withdrawing or donating of the N-substituent, their position and stereo chemistry (Blagus *et al.*, 2010). In order to expand this field of research, the title Schiff base (I) derived from an aromatic amine and 2-hydroxy-1-naphthaldehyde, has been synthesized and its crystal structure is reported herein. The Keto-amine tautomer is the favored form for this compound in solid state (Fig. 1 and Table 1). The short C9—O2 and C7—C8 bonds can be considered as C=O and C=C double bonds, respectively. The very short C10—C11 bond, suggests the presence of a significant quinoidal effect which was observed for 1-[(2-hydroxy-5-methylphenylamino)-methylene]naphthalene-2-(1*H*)-one [C=O =1.281 (2) Å; Özek *et al.*, 2004], 1-[*N*-(*p*-hydroxyphenyl)-aminomethylidene]naphthalen-2(1*H*)-one propan-1-ol hemisolvate [C=O = 1.292 (2) and 1.295 (2) Å; Odabaşoǧlu *et al.*, 2004] and 1-[(4-Acetylphenylamino)methylene]-naphthalen-2(1*H*)-one [C=O = 1.2822 (17) Å; Yüce *et al.*, 2004]. The intramolecular N1—H1···O2 hydrogen bond (Table2) stabilizes this crystallographic structure in solid state. The title compound prepared by the condensation of 4-aminophenol and 2-hydroxy-1-naphthaldehyde crystallizes in the chiral space group P212121. The crystal is photochromic in the solid state (Ünver *et al.*, 2002; Blagus *et al.*, 2010). The dihedral angle between the planes defined by O(1)—C(1)—C(2)—C(3)—C(4)—C(5)—C(6)—N(1) and C(7)—C(8)—C(9)—C(10)—C(11)—C(12)—C(13)—C(14)—C(15)—C(16)—C(17) is equal to 14.79 (7)°. The small value of bond N1—C7 (1.309 (3) Å) in comparison to bond N1—C1 (1.414 (3) Å) results in a significant change in the bond angle C1—N1—C7 of 125.97 (18)°.

### 2. Experimental

The compound is prepared by condensation of 4-aminophenol with 2-hydroxy-1-naphthaldehyde. To an ethanol solution (5 ml) of (0.109 g, 1 mmol) of 4-aminophenol was slowly added a ethanol solution (5 ml) of 2-hydroxy-1-naphthaldehyde (0.172 g, 1 mmol). The mixture was stirred under a nitrogen atmosphere and refluxed for 5 h. The red precipitate was collected by filtration and recrystallized from heated ethanoloic solution to yield red needles.

### 3. Refinement

Excepted for those attached to N atoms, witch were freely refined, all H atoms treated using a riding model with a C—H distance of 0.93 Å for aromatic H atoms. H atoms attached to the N and O atoms were located in a difference map and refined freely [N—H = 0.98 (3) Å and O—H = 0.89 (3) Å]

### Crystal data

**Table:**

C_17_H_13_NO_2_	*D*_x_ = 1.316 Mg m^−^^3^
*M**_r_* = 263.28	Mo *K*α radiation, λ = 0.71073 Å
Orthorhombic, *P*2_1_2_1_2_1_	Cell parameters from 2615 reflections
*a* = 6.1997 (7) Å	θ = 2.5–21.5°
*b* = 12.9145 (15) Å	µ = 0.09 mm^−^^1^
*c* = 16.5910 (19) Å	*T* = 296 K
*V* = 1328.4 (3) Å^3^	Needle, red
*Z* = 4	0.2 × 0.05 × 0.03 mm
*F*(000) = 552	

### Data collection

**Table:**

Bruker SMART APEXII CCD diffractometer	1445 reflections with *I* > 2σ(*I*)
Graphite monochromator	*R*_int_ = 0.038
ω scans	θ_max_ = 27.6°, θ_min_ = 2°
Absorption correction: multi-scan (*SADABS*; Bruker, 2004)	*h* = −8→8
*T*_min_ = 0.685, *T*_max_ = 0.746	*k* = −16→16
13010 measured reflections	*l* = −21→21
1791 independent reflections	

### Refinement

**Table:**

Refinement on *F*^2^	Primary atom site location: structure-invariant direct methods
Least-squares matrix: full	Secondary atom site location: difference Fourier map
*R*[*F*^2^ > 2σ(*F*^2^)] = 0.036	Hydrogen site location: inferred from neighbouring sites
*wR*(*F*^2^) = 0.099	H atoms treated by a mixture of independent and constrained refinement
*S* = 1.11	*w* = 1/[σ^2^(*F*_o_^2^) + (0.0458*P*)^2^ + 0.116*P*] where *P* = (*F*_o_^2^ + 2*F*_c_^2^)/3
1791 reflections	(Δ/σ)_max_ < 0.001
189 parameters	Δρ_max_ = 0.13 e Å^−^^3^
0 restraints	Δρ_min_ = −0.13 e Å^−^^3^

### Special details

**Table:**

Geometry. All e.s.d.'s (except the e.s.d. in the dihedral angle between two l.s. planes) are estimated using the full covariance matrix. The cell e.s.d.'s are taken into account individually in the estimation of e.s.d.'s in distances, angles and torsion angles; correlations between e.s.d.'s in cell parameters are only used when they are defined by crystal symmetry. An approximate (isotropic) treatment of cell e.s.d.'s is used for estimating e.s.d.'s involving l.s. planes.
Refinement. Refinement of *F*^2^ against ALL reflections. The weighted *R*-factor *wR* and goodness of fit *S* are based on *F*^2^, conventional *R*-factors *R* are based on *F*, with *F* set to zero for negative *F*^2^. The threshold expression of *F*^2^ > σ(*F*^2^) is used only for calculating *R*-factors(gt) *etc*. and is not relevant to the choice of reflections for refinement. *R*-factors based on *F*^2^ are statistically about twice as large as those based on *F*, and *R*- factors based on ALL data will be even larger.

### Fractional atomic coordinates and isotropic or equivalent isotropic displacement parameters (Å^2^)

**Table:**

	*x*	*y*	*z*	*U*_iso_*/*U*_eq_	
N1	0.2416 (3)	0.52850 (13)	0.64329 (11)	0.0426 (4)	
C1	0.0629 (3)	0.59631 (15)	0.63842 (12)	0.0404 (4)	
C6	−0.0379 (4)	0.62556 (18)	0.70911 (12)	0.0473 (5)	
H6A	0.0104	0.5992	0.7581	0.057*	
O2	0.4696 (3)	0.40484 (13)	0.72890 (9)	0.0576 (5)	
C7	0.3805 (4)	0.50983 (16)	0.58565 (12)	0.0428 (5)	
H7A	0.3604	0.5432	0.5365	0.051*	
O1	−0.4464 (3)	0.80231 (15)	0.63093 (10)	0.0715 (6)	
C3	−0.1847 (4)	0.70162 (17)	0.56461 (13)	0.0500 (6)	
H3A	−0.2354	0.7265	0.5155	0.06*	
C4	−0.2810 (4)	0.73383 (16)	0.63555 (13)	0.0481 (5)	
C8	0.5570 (4)	0.44303 (16)	0.59304 (12)	0.0412 (5)	
C13	0.7034 (4)	0.42826 (15)	0.52628 (12)	0.0434 (5)	
C2	−0.0154 (4)	0.63339 (16)	0.56591 (12)	0.0445 (5)	
H2B	0.0471	0.6119	0.5178	0.053*	
C10	0.7924 (4)	0.33537 (17)	0.67694 (16)	0.0557 (6)	
H10A	0.8236	0.3041	0.7261	0.067*	
C12	0.8923 (4)	0.36913 (17)	0.53822 (15)	0.0503 (6)	
C5	−0.2098 (4)	0.69353 (18)	0.70775 (13)	0.0526 (6)	
H5A	−0.2777	0.7121	0.7556	0.063*	
C9	0.5988 (4)	0.39470 (16)	0.66886 (13)	0.0464 (5)	
C17	1.0411 (5)	0.3575 (2)	0.47485 (17)	0.0664 (7)	
H17A	1.1654	0.3186	0.4831	0.08*	
C11	0.9298 (4)	0.32388 (18)	0.61540 (16)	0.0588 (6)	
H11A	1.0542	0.285	0.6232	0.071*	
C14	0.6711 (4)	0.47165 (18)	0.44906 (12)	0.0533 (6)	
H14A	0.546	0.5092	0.4388	0.064*	
C15	0.8204 (5)	0.4596 (2)	0.38897 (15)	0.0621 (7)	
H15A	0.7969	0.4903	0.339	0.075*	
C16	1.0062 (5)	0.4021 (2)	0.40172 (17)	0.0709 (8)	
H16A	1.1065	0.3942	0.3605	0.085*	
H2A	0.279 (5)	0.4942 (19)	0.6938 (15)	0.073 (8)*	
H1A	−0.468 (5)	0.834 (2)	0.6777 (19)	0.095 (10)*	

### Atomic displacement parameters (Å^2^)

**Table:**

	*U*^11^	*U*^22^	*U*^33^	*U*^12^	*U*^13^	*U*^23^
N1	0.0417 (10)	0.0478 (10)	0.0382 (9)	−0.0024 (8)	0.0015 (8)	0.0029 (8)
C1	0.0408 (11)	0.0415 (10)	0.0389 (10)	−0.0046 (9)	0.0015 (10)	−0.0003 (9)
C6	0.0496 (12)	0.0583 (13)	0.0340 (10)	0.0034 (12)	−0.0005 (10)	0.0025 (9)
O2	0.0603 (10)	0.0677 (10)	0.0449 (8)	−0.0001 (9)	0.0016 (8)	0.0134 (7)
C7	0.0435 (11)	0.0460 (11)	0.0389 (10)	−0.0051 (10)	0.0008 (9)	0.0029 (9)
O1	0.0824 (14)	0.0850 (13)	0.0471 (10)	0.0380 (12)	−0.0090 (10)	−0.0152 (9)
C3	0.0622 (14)	0.0518 (12)	0.0360 (10)	0.0058 (12)	−0.0034 (11)	−0.0019 (9)
C4	0.0522 (13)	0.0480 (11)	0.0440 (11)	0.0067 (11)	−0.0031 (11)	−0.0082 (10)
C8	0.0411 (11)	0.0416 (10)	0.0410 (10)	−0.0048 (10)	−0.0005 (9)	0.0000 (8)
C13	0.0437 (12)	0.0409 (10)	0.0457 (11)	−0.0070 (10)	−0.0002 (10)	−0.0062 (9)
C2	0.0538 (13)	0.0461 (11)	0.0336 (10)	0.0009 (10)	0.0036 (10)	−0.0014 (9)
C10	0.0585 (15)	0.0498 (13)	0.0587 (14)	0.0003 (12)	−0.0100 (13)	0.0093 (11)
C12	0.0467 (13)	0.0414 (11)	0.0626 (14)	−0.0014 (10)	0.0000 (11)	−0.0095 (10)
C5	0.0567 (14)	0.0665 (14)	0.0348 (10)	0.0083 (13)	0.0035 (11)	−0.0059 (10)
C9	0.0483 (12)	0.0444 (11)	0.0464 (11)	−0.0071 (11)	−0.0035 (11)	0.0025 (9)
C17	0.0545 (15)	0.0622 (15)	0.0825 (19)	0.0053 (14)	0.0095 (15)	−0.0211 (14)
C11	0.0522 (14)	0.0492 (13)	0.0748 (16)	0.0047 (12)	−0.0093 (14)	−0.0009 (11)
C14	0.0525 (13)	0.0622 (14)	0.0451 (12)	−0.0027 (12)	0.0023 (11)	−0.0043 (10)
C15	0.0671 (16)	0.0740 (16)	0.0452 (12)	−0.0105 (15)	0.0096 (12)	−0.0115 (12)
C16	0.0662 (18)	0.0805 (18)	0.0659 (16)	−0.0034 (16)	0.0202 (15)	−0.0203 (15)

### Geometric parameters (Å, º)

**Table:**

N1—C7	1.309 (3)	C13—C14	1.413 (3)
N1—C1	1.414 (3)	C13—C12	1.412 (3)
N1—H2A	0.98 (3)	C2—H2B	0.93
C1—C6	1.382 (3)	C10—C11	1.338 (3)
C1—C2	1.383 (3)	C10—C9	1.431 (3)
C6—C5	1.381 (3)	C10—H10A	0.93
C6—H6A	0.93	C12—C17	1.407 (3)
O2—C9	1.285 (3)	C12—C11	1.427 (3)
C7—C8	1.399 (3)	C5—H5A	0.93
C7—H7A	0.93	C17—C16	1.360 (4)
O1—C4	1.356 (3)	C17—H17A	0.93
O1—H1A	0.89 (3)	C11—H11A	0.93
C3—C2	1.371 (3)	C14—C15	1.370 (3)
C3—C4	1.384 (3)	C14—H14A	0.93
C3—H3A	0.93	C15—C16	1.386 (4)
C4—C5	1.379 (3)	C15—H15A	0.93
C8—C9	1.428 (3)	C16—H16A	0.93
C8—C13	1.445 (3)		
			
C7—N1—C1	125.97 (18)	C11—C10—C9	121.5 (2)
C7—N1—H2A	112.8 (16)	C11—C10—H10A	119.3
C1—N1—H2A	121.1 (16)	C9—C10—H10A	119.3
C6—C1—C2	119.00 (19)	C17—C12—C13	119.8 (2)
C6—C1—N1	118.38 (17)	C17—C12—C11	121.4 (2)
C2—C1—N1	122.62 (18)	C13—C12—C11	118.8 (2)
C5—C6—C1	120.61 (19)	C6—C5—C4	120.1 (2)
C5—C6—H6A	119.7	C6—C5—H5A	120
C1—C6—H6A	119.7	C4—C5—H5A	120
N1—C7—C8	124.34 (19)	O2—C9—C8	121.7 (2)
N1—C7—H7A	117.8	O2—C9—C10	120.32 (19)
C8—C7—H7A	117.8	C8—C9—C10	118.0 (2)
C4—O1—H1A	112 (2)	C16—C17—C12	121.1 (3)
C2—C3—C4	120.7 (2)	C16—C17—H17A	119.4
C2—C3—H3A	119.7	C12—C17—H17A	119.4
C4—C3—H3A	119.7	C10—C11—C12	122.4 (2)
O1—C4—C5	122.51 (19)	C10—C11—H11A	118.8
O1—C4—C3	118.31 (19)	C12—C11—H11A	118.8
C5—C4—C3	119.2 (2)	C15—C14—C13	121.3 (2)
C7—C8—C9	119.26 (19)	C15—C14—H14A	119.4
C7—C8—C13	120.38 (18)	C13—C14—H14A	119.4
C9—C8—C13	120.25 (19)	C14—C15—C16	120.8 (2)
C14—C13—C12	117.3 (2)	C14—C15—H15A	119.6
C14—C13—C8	123.6 (2)	C16—C15—H15A	119.6
C12—C13—C8	119.01 (19)	C17—C16—C15	119.7 (3)
C3—C2—C1	120.35 (19)	C17—C16—H16A	120.2
C3—C2—H2B	119.8	C15—C16—H16A	120.2
C1—C2—H2B	119.8		

### Hydrogen-bond geometry (Å, º)

**Table:**

*D*—H···*A*	*D*—H	H···*A*	*D*···*A*	*D*—H···*A*
N1—H2*A*···O2	0.98 (3)	1.75 (3)	2.563 (2)	138 (2)
O1—H1*A*···O2^i^	0.89 (3)	1.80 (3)	2.680 (2)	171 (3)

Symmetry code: (i) −*x*, *y*+1/2, −*z*+3/2.

## References

[bb1] Alpaslan, G., Macit, M., Erdönmez, A. & Büyükgüngör, O. (2011). *Struct. Chem.* **22**, 681–690.

[bb2] Blagus, A., Cinčić, D., Friščić, T., Kaitner, B. & Stilinović, V. (2010). *Maced. J. Chem. Chem. Eng.* **29**, 117–138.

[bb3] Bruker (2004). *APEX2*, *SAINT* and *SADABS* Bruker AXS Inc., Madison, Wisconsin, USA.

[bb4] Farrugia, L. J. (2012). *J. Appl. Cryst.* **45**, 849–854.

[bb5] Odabaşoǧlu, M., Albayrak, Ç. & Büyükgüngör, O. (2004). *Acta Cryst.* E**60**, o142–o144. 10.1107/S0108270104010832 15178879

[bb6] Özek, A., Yüce, S., Albayrak, Ç., Odabaşoğlu, M. & Büyükgüngör, O. (2004). *Acta Cryst.* E**60**, o828–o830.

[bb7] Sheldrick, G. M. (2008). *Acta Cryst.* A**64**, 112–122. 10.1107/S0108767307043930 18156677

[bb8] Ünver, H., Kendi, E., Güven, K. & Durlu, T. (2002). *Z. Naturforsch. Teil B*, **57**, 685–690.

[bb9] Westrip, S. P. (2010). *J. Appl. Cryst.* **43**, 920–925.

[bb10] Yüce, S., Özek, A., Albayrak, Ç., Odabaşoğlu, M. & Büyükgüngör, O. (2004). *Acta Cryst.* E**60**, o1217–o1218.

